# Enriched Expression of Neutral Sphingomyelinase 2 in the Striatum is Essential for Regulation of Lipid Raft Content and Motor Coordination

**DOI:** 10.1007/s12035-017-0784-z

**Published:** 2017-10-17

**Authors:** Laura Hui-Ru Tan, Angela Jin-Rong Tan, Yu-Ying Ng, John Jia-En Chua, Wee-Siong Chew, Sneha Muralidharan, Federico Torta, Bamaprasad Dutta, Siu Kwan Sze, Deron R. Herr, Wei-Yi Ong

**Affiliations:** 1 Department of Anatomy, National University of Singapore, Singapore, 119260 Singapore; 2 Neurobiology and Ageing Research Programme, National University of Singapore, Singapore, 119260 Singapore; 3 Department of Physiology, National University of Singapore, Singapore, 119260 Singapore; 4 Institute of Molecular and Cell Biology, Agency for Science, Technology and Research (A*STAR), Singapore, 138673 Singapore; 5 Department of Pharmacology, National University of Singapore, Singapore, 119260 Singapore; 6 Department of Biological Sciences, National University of Singapore, Singapore, 119260 Singapore; 7 Department of Biochemistry, National University of Singapore, Singapore, 119260 Singapore; 8 School of Biological Sciences, Nanyang Technological University, Singapore, 637551 Singapore

**Keywords:** nSMase2, Motor function, Startle reflex, Lipid rafts, GW4869, Antisense knockdown, Membrane lipids, Microdomains, Lipidomics, Proteomics, Sphingolipids

## Abstract

Sphingomyelinases are a family of enzymes that hydrolyze sphingomyelin to generate phosphocholine and ceramide. The brain distribution and function of neutral sphingomyelinase 2 (nSMase2) were elucidated in this study. nSMase2 mRNA expression was greatest in the striatum, followed by the prefrontal cortex, hippocampus, cerebellum, thalamus, brainstem, and olfactory bulb. The striatum had the highest level of nSMase2 protein expression, followed by the prefrontal cortex, thalamus, hippocampus, brainstem, and cerebellum. Dense immunolabeling was observed in the striatum, including the caudate-putamen, while moderately dense staining was found in the olfactory bulb and cerebral neocortex. Electron microscopy of the caudate-putamen showed nSMase2 immunoreaction product was present in small diameter dendrites or dendritic spines, that formed asymmetrical synapses with unlabeled axon terminals containing small round vesicles; and characteristics of glutamatergic axons. Lipidomic analysis of the striatum showed increase in long chain sphingomyelins, SM36:1 and SM38:1 after inhibition of nSMase activity. Quantitative proteomic analysis of striatal lipid raft fraction showed many proteins were downregulated by more than 2-fold after inhibition or antisense knockdown of nSMase; consistent with the notion that nSMase2 activity is important for aggregation or clustering of proteins in lipid rafts. Inhibition or antisense knockdown of nSMase2 in the caudate-putamen resulted in motor deficits in the rotarod and narrow beam tests; as well as decreased acoustic startle and improved prepulse inhibition of the startle reflex. Together, results indicate an important function of nSMase2 in the striatum.

**Electronic supplementary material:**

The online version of this article (10.1007/s12035-017-0784-z) contains supplementary material, which is available to authorized users.

## Introduction

Sphingomyelinases (SMases) belong to a class of enzymes that hydrolyze sphingomyelin to generate phosphocholine and ceramide. They play key roles in sphingomyelin breakdown that results in altered neural membrane compositions and the generation of ceramide, a well-established modulator of various important cellular signaling pathways regulating cell differentiation, proliferation, cell survival, and cell death [1–3]. They are categorized according to their pH level at which their activity is optimal and include lysosomal acid sphingomyelinase (A-SMase), secreted zinc-dependent acid sphingomyelinase (S-SMase), magnesium-dependent neutral sphingomyelinase (nSMase), magnesium-independent neutral sphingomyelinase, and alkaline sphingomyelinase. Of these, A-SMase and nSMase are thought to be the major players in stress-induced ceramide production [4, 5]. Four neutral nSMases have been identified, namely, nSMase1, nSMase2, nSMase3, and MA-nSMase (mitochondrial-associated nSMase). They require a neutral pH and divalent cations such as Mg^2+^ or Mn^2+^ to specifically hydrolyze the phosphocholine-headgroup from sphingomyelin [6, 7]. With the exception of nSMase3, nSMases possess a DNase I-type catalytic core, suggesting a common mechanism for sphingomyelin catalysis [4].

A comparison of the three nSMase isoforms is presented in Table 1 [8–16]. nSMase2 is a 655 amino acid protein with a molecular weight of 71 kDa. It appears to be the most prominent and well-researched isoform. nSMase2 is localized at the Golgi apparatus and plasma membranes in certain cell lines [17, 18]. Various factors such as TNF-α, PMA [19], and H_2_O_2_ [4] trigger nSMase2 to translocate from the Golgi apparatus to the plasma membrane. nSMase2 is involved in the modulation of dendritic spine size [20], hippocampal neurite outgrowth, and synaptogenesis [21]. Pharmacological inhibition or genetic mutation of nSMase2 prevented TNFα-induced generation of ceramide, phosphorylation of NR1 subunits, clustering of NR1, enhancement of *N*-methyl-d-aspartate (NMDA)-evoked calcium flux and excitatory post-synaptic currents in cultured hippocampal neurons [22]. nSMase2 inhibition also increased PSD-95, increased the number of AMPA receptors, and impaired spatial and episodic-like memory in mice [23]. Inhibition of nSMase2 via treatment with nSMase2 siRNA or nSMase inhibitor GW4869 decreases ceramide production and reduces dopamine reuptake in PC12 cells, while addition of C6 ceramide increases dopamine uptake [24]. nSMase2 may also have a role in Alzheimer’s disease. It is activated by Aβ and leads to accumulation of ceramide in neurons, oligodendrocytes and cerebral endothelial cells, and apoptosis [25–27].

**Table 1:** Comparison between the three nSMase isoforms. Based on data from references [8–16]

	nSMase1	nSMase2	nSMase3
Optimal pH	7.4		
Molecular weight (kDa)	47.5	71	97.8
Substrate specificity	Lyso-platelet activating factor	Sphingomyelin	
Inhibitors	3-*O*-Methyl-sphingomyelin (3-OMS), kotylostatin, manumycin A		
	No NSM1-specific inhibitor	GW4869, cambinol	Scyphostatin
Cellular localization	Endoplasmic reticulum, Golgi apparatus, and/or nuclear matrix	Golgi apparatus and plasma membrane	Endoplasmic reticulum and Golgi apparatus
Expression	Multiple tissues	Highest expression in the brain	Highest expression in striated muscle and heart muscle

**Table 2:** Acoustic startle reflex. Summary of different trials. The session consisted of 32 discrete trials, which were conducted in pseudorandom order. The prepulse-to-pulse interval was set at 100 ms.

Trial	Number of trials	Intensities (dB)	Duration
Acclimation/no stimulus	–	65	5 min
Pulse	17	120	40 ms
Prepulse + pulse	5	69 + 120	20 ms + 40 ms
	5	73 + 120	
	5	77 + 120	

Although much attention has been placed on the functional and physiological roles of nSMases in vitro, relatively little is known about the normal expression and function of nSMase2 in the brain. Previous studies have shown high level of nSMase2 activity in the parietal cortex and corpus striatum of the rat brain [28]. In this study, we identified the striatum as the brain region that has among the highest expression of nSMase2, and carried out intrastriatal inhibition of nSMase2 to establish its effect on lipid and protein profiles and behavior. Results demonstrate an important role of nSMase2 in motor function and sensorimotor gating.

## Materials and Methods

### Chemicals

The nSMase inhibitor GW4869 was purchased from Santa Cruz (Santa Cruz Biotechnology, Santa Cruz, USA) and was diluted in vehicle (saline). Antisense and scrambled sense nSMase2 oligonucleotides were purchased from Exiqon (Exiqon A/S, Denmark) of 20 nmol concentration. The sequences were 5′ GTAGAAAATCGTGACT 3′ for the antisense and 5′ TGATAAAATCGTGGCA 3′ for scrambled sense oligonucleotide. Both antisense and scrambled sense oligonucleotides were reconstituted in 10 μl of nuclease-free water.

### Animals

Adult male Wistar rats (250–300 g) were procured from InVivos Singapore and housed in temperature-controlled (23 ± 1 °C), individually ventilated cages on a 12-h light/12-h dark cycle (7 a.m.–7 p.m.) with access to food and water. Rats were acclimatized for 4 days prior to the start of experiments. All procedures were in accordance with the Principles of Laboratory Animal Care and approved by the Institutional Animal Care and Use Committee of the National University of Singapore.

### Stereotaxic Injections

Anesthesia was induced and maintained in rats using the inhalational anesthetic isoflurane (Sigma-Aldrich, St Louis, USA). These were then mounted on a stereotaxic frame (Stoelting, Wood Dale, USA) and the bregma exposed via a midline incision on the scalp. Small craniotomies were performed over the injection sites: 1.0 mm anterior and 3.0 mm lateral to the bregma on both sides, and 5.0 mm from the surface of the cortex. These coordinates correspond to the caudate-putamen of the striatum which was determined using the rat brain atlas of Paxinos and Watson [29]. GW4869 or saline, or antisense or scrambled sense oligonucleotides were injected into the striatum bilaterally. The volume injected at each site was 2 μl and the injection rate was 4 min per injection.

### Real-Time Reverse Transcriptase Polymerase Chain Reaction

Six adult male Wistar rats were used for this part of the study. They were deeply anesthetized with intraperitoneal injection of ketamine and xylazine cocktail (prepared with 7.5 ml ketamine (75 mg/kg), 5 ml xylazine (10 mg/kg), and 7.5 ml 0.9% sodium chloride solution) and decapitated. The olfactory bulb, prefrontal cortex, striatum, thalamus, cortex 1 (which includes the primary and secondary motor cortex and primary somatosensory cortex), cortex 2 (which includes the parietal association cortex and secondary auditory cortex), hippocampus, cerebellum, and brainstem were quickly dissected, immersed in RNAlater® solution (Ambion, TX, USA), and snap frozen in liquid nitrogen. TRizol reagent (Invitrogen, CA, USA) was used to extract total RNA, according to the manufacturer’s protocol. Purification of RNA was performed using RNeasy® Mini Kit (Qiagen, Inc., CA, USA). Reverse transcription was done using the High-Capacity cDNA Reverse Transcription Kit (Applied Biosystems, CA, USA). The reaction conditions were as follows: 25 °C for 10 min, 37 °C for 120 min, and 85 °C for 5 s. Real-time PCR amplification was performed using 7500 Real-Time PCR system (Applied Biosystems, CA, USA), TaqMan® Universal PCR Master Mix (Applied Biosystems, CA, USA), and TaqMan® Gene Expression Assay Probes for A-SMase, nSMase1, nSMase2, nSMase3, and β-actin (Applied Biosystems, CA, USA) according to the manufacturer’s instructions. The PCR conditions were as follows: incubation at 50 °C for 2 min and 95 °C for 10 min followed by 40 cycles of 95 °C for 15 s and 60 °C for 1 min. All reactions were performed in triplicates. The threshold cycle (CT) was determined based on the number of cycles in which the reporter fluorescence emission exceeded the preset threshold level. Amplified transcripts were calculated using the comparative CT method against the standard curve and the formula for relative fold change = 2^−∆∆CT^ [30]. Relative quantification of each of the nSMase isoforms was performed using a standard curve for each target.

### Western Blot Analysis

Four adult Wistar rats were used for this part of the study. They were anesthetized deeply and sacrificed by decapitation. Different parts of the rat brain such as the olfactory bulb, prefrontal cortex, striatum, thalamus, cortex 1, cortex 2, hippocampus, cerebellum, and brain stem were dissected and homogenized using a Tissue Tearor™ (Biospec, OK, USA) in T-Per® Tissue Protein Extraction Solution containing 1% Halt™ protease inhibitor and 1% EDTA solution (Thermo Fisher Scientific, IL, USA). The homogenates were centrifuged at 12,000*g*, and the protein concentration in the supernatant was calculated using the Bio-Rad protein assay kit (Bio-Rad Laboratories, CA, USA). Total proteins were resolved in 15% SDS polyacrylamide gels under reducing conditions and electrotransferred to polyvinylidene difluoride (PVDF) membranes (Amersham Pharmacia Biotech, Little Chalfont, UK). This was incubated with 5% skim milk in 0.1% TBS-Tween for 1 h to block non-specific binding sites. The PVDF membrane was then incubated overnight with a rabbit polyclonal antibody to nSMase2, diluted 1:1600 dilution in 5% skim milk in 0.1% TBS-Tween at 4 °C. The antibody was raised against a region within amino acids 461 and 655 at the C-terminus of nSMase2 of human origin (Santa Cruz Biotechnology, Santa Cruz, USA). The membrane was incubated with horseradish peroxidase-conjugated anti-rabbit IgG (ThermoFisher Scientific, MA, USA) for 1 h at room temperature. Labeled protein was visualized with an enhanced chemiluminescence kit according to the manufacturer’s instructions (Pierce, Rockford, IL, USA). Intensities of the bands were quantified using densitometric analyses. Western blots were also used to verify knockdown of nSMase2 protein by antisense oligonucleotide. Rats used in the rotarod and balance beam tests were anesthetized deeply and sacrificed by decapitation. The striatum was dissected from these rats and analyzed by Western blots to check knockdown efficiency.

### Immunohistochemistry

Six adult Wistar rats were used for this portion of the study. They were anesthetized deeply and perfused through the left cardiac ventricle with a mixture of 4% paraformaldehyde and 0.1% glutaraldehyde in 0.1 M phosphate buffer (pH 7.4). The brains were removed and sectioned coronally at 100 μm using a vibratome. Sections were washed for 3 h with phosphate-buffered saline (PBS) and incubated overnight at 4 °C with an affinity-purified rabbit polyclonal antibody to nSMase2 (Santa Cruz Biotechnology, Santa Cruz, USA), diluted 1:200 in PBS. They were then incubated for 1 h in a 1:200 dilution of biotinylated horse anti-rabbit IgG (Vector, Burlingame, CA, USA), reacted for 1 h with avidin-biotinylated horseradish peroxidase complex, and treated for 5 min in 0.05% 3, 3-diaminobenzidine tetrahydrochloride solution in Tris buffer containing 0.05% hydrogen peroxide. Some of the sections were mounted on glass slides, counterstained with methyl green, and coverslipped, while the remaining sections were processed for electron microscopy.

### Electron Microscopy

Immunostained sections of the striatum were subdissected into smaller portions and stained with 1% osmium tetroxide for 1 h. They were dehydrated in an ascending series of 25, 50, 75, and 100% ethanol and acetone, and embedded in Araldite. Thin sections were obtained from the first 5 μm of the immunostained sections, mounted on Formvar-coated copper grids, and stained with lead citrate. They were viewed using a JEOL 1010EX electron microscope (JEOL, Japan).

### Lipidomic Analysis

Rats that have completed the rotarod and balance beam tests (see below) were used for this part of the study, with six rats in each group that received GW4869 or saline injection to the caudate-putamen. These were deeply anesthetized and sacrificed by decapitation, and the striatum dissected out and snap frozen in liquid nitrogen. The tissue was thawed, and weighed, and lipids were extracted and analyzed for lipidomics as previously described [31]. Briefly, the tissue was transferred to Omni tubes containing 50 mg of 1.4-mm ceramic beads. One milliliter of BUME (butanol: methanol 1:1) mixture containing 1.07 nmol/ml of 12:0 SM (860583, Avanti Polar Lipids, USA) and 0.18 nmol/ml of C17 ceramide (860517, Avanti Polar Lipids, USA) standards was added to around 30 mg of wet tissue that was then homogenized for few seconds using an Omni bead mill homogenizer. The homogenized tissue was sonicated for 30 min and centrifuged at 14,000*g* to precipitate the protein phase. The supernatant, containing the extracted lipids, was analyzed by positive mode electrospray ionization (ESI) mass spectrometry (MS) using an Agilent 6495 QQQ mass spectrometer. Lipid separations were performed on a UHPLC Agilent 1290, using a reversed phase Agilent ZORBAX RRHD Eclipse Plus C18 column (95 Å, 2.1 × 100 mm, 1.8 μm) at 40 °C. Mobile phases A (40% acetonitrile in 60% water with 10 mM ammonium formate) and B (10% acetonitrile in 90% isopropanol with 10 mM ammonium formate) were mixed according to the following gradient: 20% B at 0 min, to 60% B at 2.0 min, to 100% B at 7.0 min, maintained at 100% B from 7.0 to 9.0 min, re equilibrated at 20%B from 9.01 min to 10.80 min. Lipids were measured using a dynamic MRM method. Possible significant differences were analyzed using Student's *t* test. *P* < 0.05 was considered significant.

### Proteomic Analysis of Lipid Rafts

Rats that have completed the rotarod and balance beam tests were used for this part of the study, with six rats in each group that received GW4869 or saline; or antisense or scrambled sense oligonucleotide injection to the caudate-putamen. To determine if nSMase2 was involved in the proteome of lipid rafts, a label-free quantitative proteomics approach was carried out using LC-MS/MS analysis and Protein Abundance Index (emPAI) protocol. Rats were anesthetized deeply and sacrificed by decapitation. The striatum was dissected out, and in view of the small amount of tissue, lipid rafts were isolated using the UltraRIPA kit according to the manufacturer’s instructions (BioDynamics Laboratory, Japan). This is a novel but validated small-scale platform to rapidly extract membrane proteins or membrane-associated proteins enriched in lipid rafts with a native structure and function [32]. In brief, rat striatal samples were first homogenized in conventional RIPA buffer (50 mM Tris-HCl (pH 8.0), 150 mM NaCl, 1% NP-40 alternative, 0.1% SDS, and 0.5% sodium deoxycholate) using a Tissue Tearor™ (Biospec, OK, USA). After purification of the RIPA-insoluble fraction, lipid raft proteins were extracted using another B-buffer which solubilizes the RIPA-insoluble lipid raft proteins (BioDynamics Laboratory, Japan). Lipid raft fractions from the four groups were resolved by 12.5% SDS-PAGE and stained with Coomassie brilliant blue. Each sample lane was cut separately and sliced into small pieces for destaining, washing, reduction, alkylation, and trypsin digestion using sequencing-grade modified trypsin (Promega, Madison, WI), at 37 °C overnight. Tryptic peptides were extracted with 5% acetic acid/50% ACN buffer and vacuum dried. They were reconstituted in 0.1% formic acid (FA) solution before LC-MS/MS analysis using an online Dionex UltiMate 3000 UHPLC system coupled with a Q Exactive mass spectrometer (Thermo Scientific Inc., Bremen, Germany). Each sample was injected into the LC-MS/MS for three times. The acquired raw data were converted into the mascot generic format (mgf) files using Proteome Discoverer 1.4.1.14 software (Thermo Fisher, MA) with MS2 spectrum processor for deisotoping. All the searches were performed using the in-house mascot search engine (Mascot, version 2.4.1; Matrix Science, London, UK) using UniProt Knowledgebase (UniProtKB) rat database (31,555 sequences, 17,339,165 residues) along with the reverse sequences. Carbamidomethyl at cysteine was set as a static modification and methionine oxidation, and asparagines and glutamine deamidation as dynamic modifications. Full trypsin digestion with maximum 2 missed and/or non-specific cleavages set as digestion parameter; while no. 13C of 2, 10 ppm precursor mass, and 0.02-Da fragment mass tolerance were set as other search parameters. Target-decoy search strategy with cutoff set to ≤ 1% false density rate (FDR) and proteins identified with > 2 unique peptides were used for further analysis. The emPAI value for each identified protein was calculated by Mascot during search and was used for the label-free quantification.

### Rotarod Test

Six adult male Wistar rats in each group, i.e., GW4869, saline, antisense-, or scrambled sense oligonucleotides, were used in this portion of the study. Rats underwent intrastriatal injection of GW4869 or saline 6 h before undergoing the rotarod test. The latter was carried out 6-h post injection, since the inhibitory effect of GW4869 was shown to be significantly reduced by 24 h [12]. For the oligonucleotide-injected groups, rats underwent intrastriatal injection of antisense or scrambled sense oligonucleotides before undergoing rotarod testing for 5 days. They were positioned on an accelerating rotarod cylinder and were timed based on how long they remained on the rotarod. The speed gradually increased from 4 to 40 rpm within a span of 5 min. A trial was considered to have ended when a rat fell off the rotarod cylinder or gripped the cylinder and spun for 2 successive revolutions without trying to walk on the rungs. The duration (in seconds) on the device was recorded. Rotarod test data were presented as average latency to fall (3 trials) from the rotarod. Possible significant differences were analyzed using Student's *t* test. *P* < 0.05 was considered significant.

### Beam Crossing Test

The time to cross the center 80 cm of a 1-m round beam of 2.5-cm diameter was recorded. The beam was elevated 90 cm over the ground and had to be crossed three times. Rats were positioned at one end of the beam with the animal’s home cage, bedding material, and food pellets at the opposite end. Rats were trained 2 days prior to the day of testing. During the training period, rats were encouraged to cross the beam by gentle nudging. Training trials were performed repeatedly until each animal successfully traversed the beam three times without pausing or turning around. On the day of testing, a baseline recording of three trials per rat was performed before intrastriatal injection of GW4869 or saline. Six hours after injection, rats were made to cross the beam for three trials. Trials where the rat spent more than 60 s to cross or fell from the beam were recorded as 60 s. Trials where the rat paused or turned around were repeated. The average of the trials was recorded. The same testing procedure was performed for 5 days after intrastriatal injection of antisense or scrambled sense oligonucleotide. Possible significant differences were analyzed using Student's *t* test. *P* < 0.05 was considered significant.

### Acoustic Startle Reflex Test

Startle reflex testing was conducted 6 h after surgery, using a startle chamber that contained a transparent Plexiglas tube mounted on a Plexiglas frame (SR-LAB, San Diego Instruments, San Diego, CA, USA)(Table 2). A high-frequency loudspeaker located in the chamber emits an uninterrupted background noise of 65 dB and the various acoustic stimuli. Vibrations from the Plexiglas cylinder as a result of the whole body startle response of the rats were transduced into analog signals (0–10,000-mV range) recorded by the load cell platform. The signals were digitized and analyzed using SR-LAB Startle Response Software (San Diego Instruments, San Diego, CA, USA). The protocol for measuring prepulse inhibition is based on a previous study [33]. The startle session proceeded after a 5-min acclimatization period in the tube with a background noise level of 65 dB that was sustained for the entire startle session. Animals were then exposed to a succession of 32 discrete trials comprising of 17 40-ms presentations of a 120-dB pulse (pulse-alone), five 20-ms presentations of each prepulse intensity (69, 73, 77 dB) 100 ms prior to a 40-ms presentation of a 120-dB pulse (prepulse + pulse). No-stimulus trials, whereby no acoustic pulse was delivered, were also recorded to evaluate general motor activity of the animals. The pulse and prepulse stimuli used were in the form of a sudden elevation in broadband white noise level from background (65 dB), and trials were conducted in pseudorandom order. Startle magnitude was calculated based on the pulse-alone (120 dB) trials and the prepulse + pulse trials. Percentage prepulse intensities (%PPI) for the three prepulse intensities were derived using the formula: %PPI = (pulse-alone − prepulse + pulse)/(pulse-alone) × 100%. Possible significant differences were analyzed using Student's *t* test. *P* < 0.05 was considered significant.

### TUNEL and DAPI Histochemistry

Four sets of rats injected with GW4869 or vehicle control from the acoustic startle reflex experiment were sacrificed 1 day after experiment, to determine if GW4869 inhibition of nSMase2 led to cell death. Animals were anesthetized deeply and perfused through the left cardiac ventricle with Ringer’s solution followed by 4% paraformaldehyde in 0.1 M phosphate buffer (pH 7.4). The brains were harvested and sectioned coronally at 20 μm using a cryostat. Sections were mounted on slides coated with gelatin and observed using a microscope to locate the needle track from intrastriatal injections to establish the plane for TUNEL staining. Sections were made permeable with 0.1% Triton X-100 and 0.1% sodium citrate in PBS for 10 min on ice. TUNEL staining was done with In-Situ Cell Death Detection Kit, Fluorescein (Roche Diagnostics, Basel, Switzerland) to identify DNA fragmentation, according to the manufacturer’s protocol. The nuclear counterstaining and mounting agent used was ProLong® Gold Anti-fade reagent with DAPI (Invitrogen, Waltham, USA). Positive controls were incubated with DNase I (3 U/μl) (Roche Diagnostics) for 10 min at room temperature. Sections were examined with an Olympus BX51 microscope (Olympus, Tokyo, Japan).

## Results

### Differential mRNA Expression of SMase Isoforms in the CNS

**Fig. 1:**
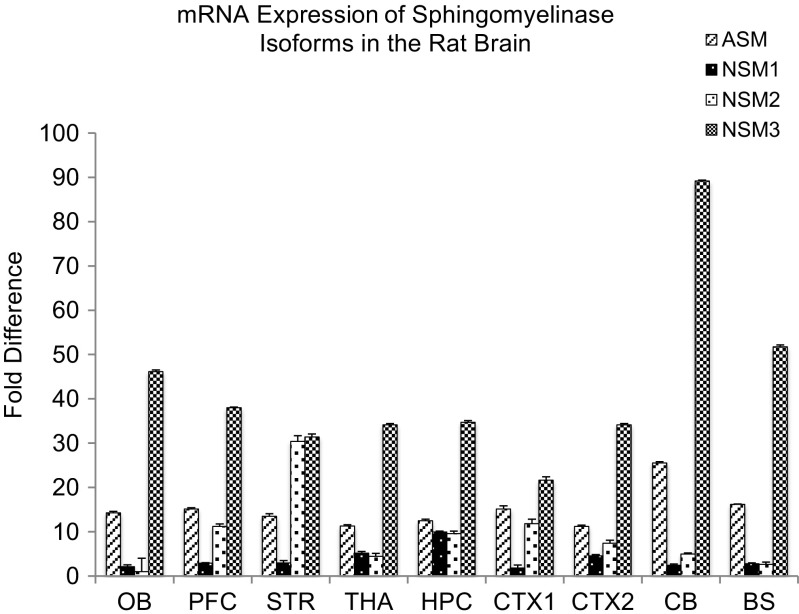
Real-time RT-PCR analyses of A-SMase (ASM), nSMase 1 (NSM1), nSMase 2 (NSM2), nSMase 3 (NSM3) mRNA distribution in various parts of the rat brain including olfactory bulb (OB), prefrontal cortex (PFC), striatum (STR), thalamus/hypothalamus (THA), hippocampus (HPC), cortex 1 (CTX1), cortex 2 (CTX2), cerebellum (CB), and brainstem (BS). Values were normalized to the lowest expressing nSMase2 in the olfactory bulb. Data represents the mean and standard error from *n* = 6 Wistar rats

The mRNA expression of different sphingomyelinase isoforms was quantified by real-time RT-PCR, to determine their relative expression in the brain. nSMase3, acid sphingomyelinase, and nSMase2 were found to have higher expression than nSMase 1 in the brain (Fig. 1). However, since other studies measuring nSMase activity have raised the possibility that nSMase3 activity may differ from that of other nSMases [34], and nSMase3 does not share any sequence homology or common catalytic core residues with other nSMase isoforms [8], nSMase2 was chosen for further analysis in this study.

### Expression of nSMase2 in Different Brain Regions at mRNA, Protein, and Cellular and Subcellular Levels

Comparison of mRNA level of nSMase2 in different regions of the CNS showed that expression was highest in the striatum, followed by the prefrontal cortex, hippocampus, cortex 1 (which includes the primary and secondary motor cortex and primary somatosensory cortex), cortex 2 (which includes the parietal association cortex and secondary auditory cortex), cerebellum, thalamus, brainstem, and olfactory bulb (Fig. 1).

**Fig. 2:**
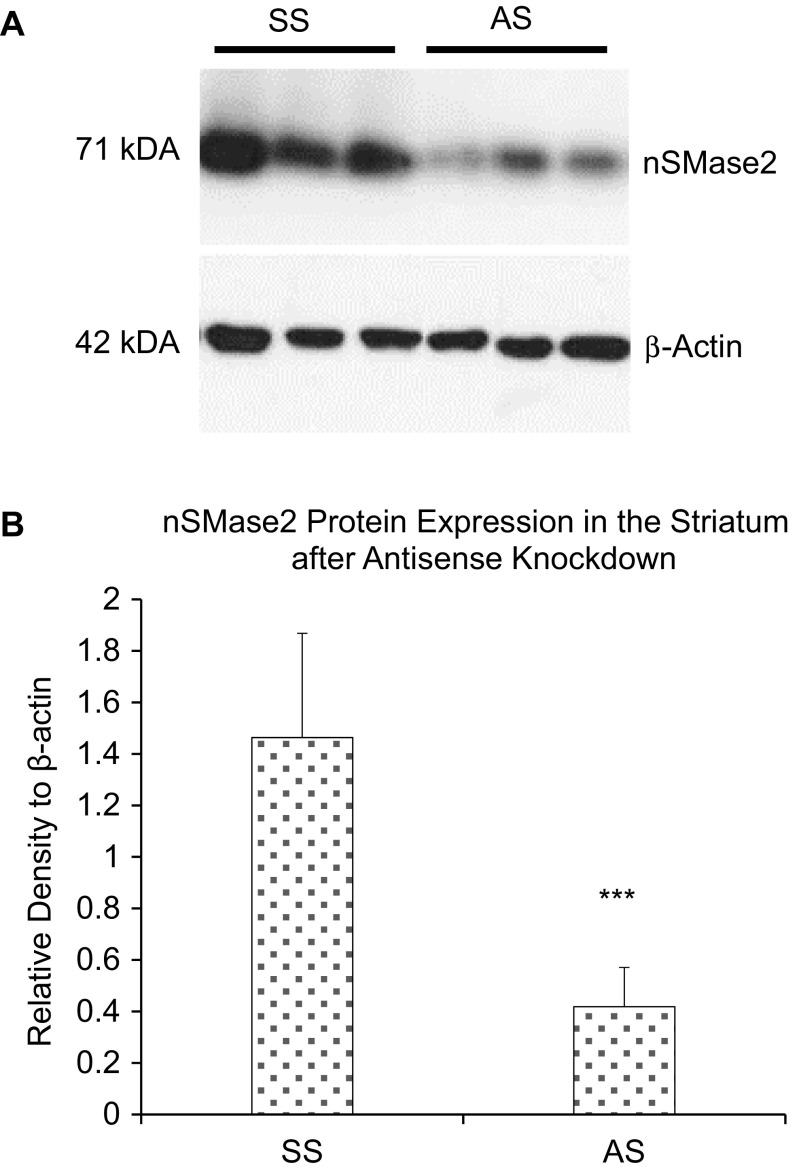
**a** Immunoblot of the striatum of adult rats injected with scrambled sense nSMase2 (SS) and antisense nSMase2 (AS). Antisense knockdown of nSMase2 resulted in reduced density of the 71-kDa band, indicating effectiveness of the knockdown, as well as specificity of the antibody. **b** Densitometric analysis of nSMase2 band intensities of SS- and AS-injected rats, normalized to β-actin. Data represents the mean and standard error from *n* = 6 Wistar rats in each group. Each bar in the diagram indicates mean + SEM

**Fig. 3:**
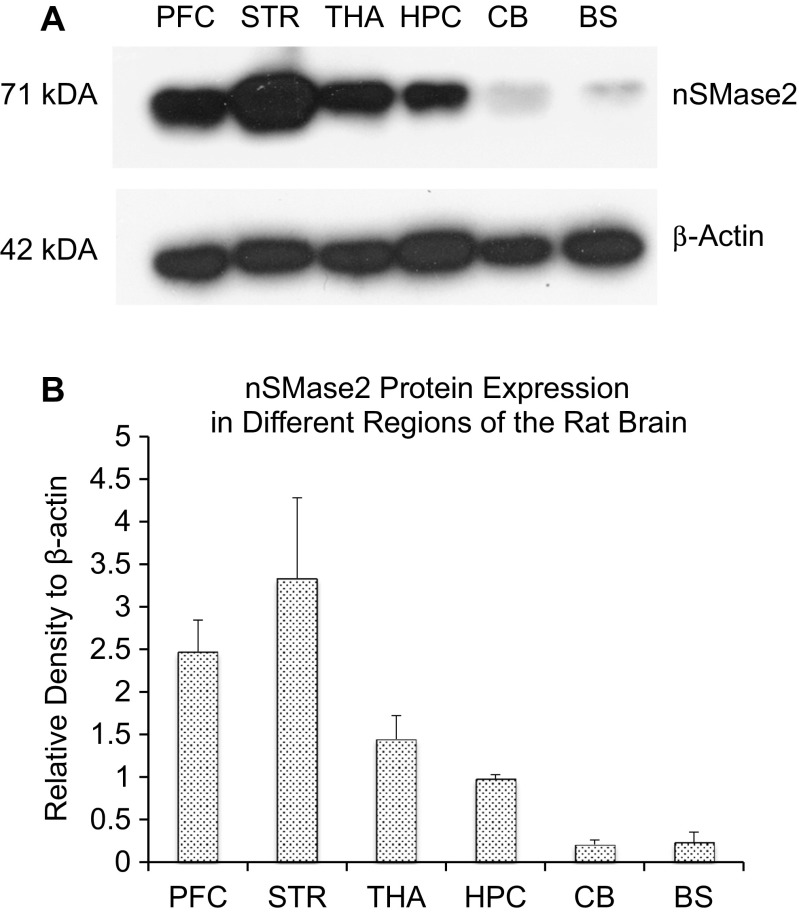
**a** Immunoblot of adult rats in selected parts of the rat brain including the prefrontal cortex (PFC), striatum (STR), thalamus (THA), hippocampus (HPC), cerebellum (CB), and brainstem (BS). The striatum was found to have the highest nSMase2 protein expression, followed by the prefrontal cortex, thalamus, hippocampus, brainstem, and cerebellum. **b** Densitometric analysis of nSMase2 band intensities, normalized to β-actin. Data represents the mean and standard error from *n* = 4 Wistar rats. Each bar in the diagram indicates mean + SEM

The protein expression of nSMase2 in different brain regions was then determined. Western blotting with nSMase2 antibody detected a single 71-kDa band in the adult rat brain, consistent with the expected molecular weight of full length nSMase2 protein. Antisense knockdown of nSMase2 resulted in reduced density of the 71-kDa band, indicating effectiveness of the knockdown, as well as specificity of the antibody (Fig. 2). The striatum had the highest level of nSMase2 protein expression, followed by the prefrontal cortex, thalamus, hippocampus, brainstem, and cerebellum (Fig. 3a, b).

**Fig. 4:**
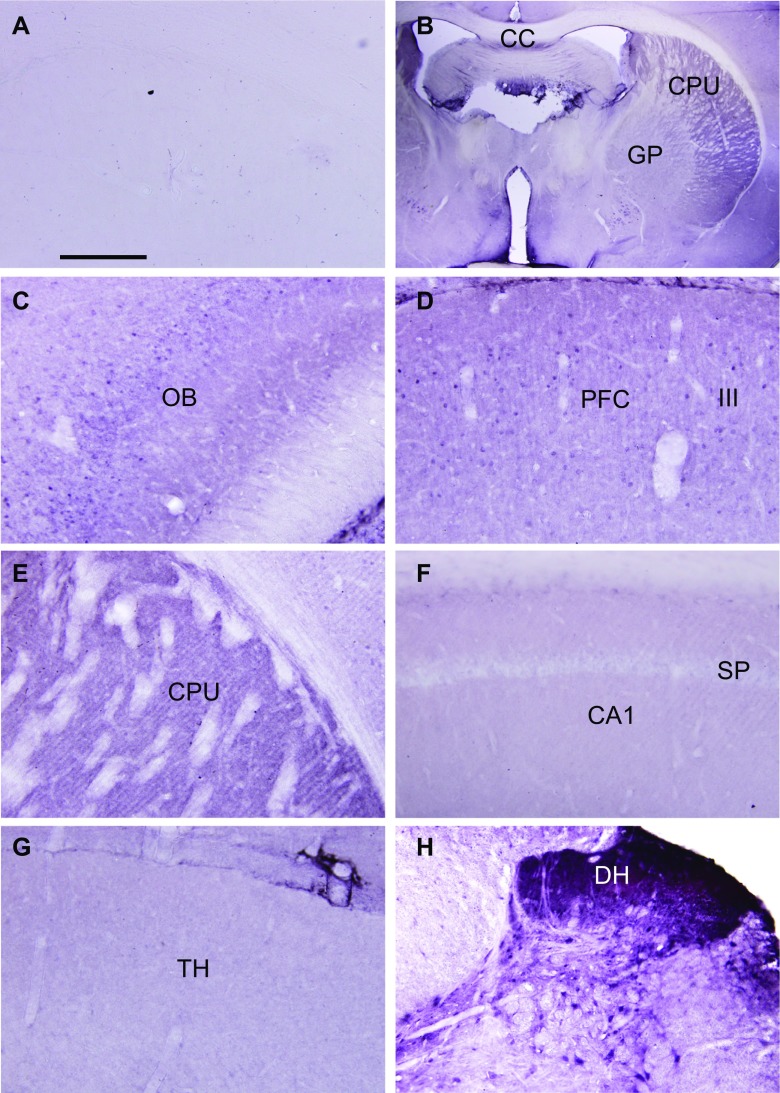
Light micrographs of control and nSMase2-immunolabeled sections of the rat CNS. **a** Control section from the striatum which had been incubated with PBS instead of primary antibody, showing absence of staining. **b** Low magnification showing the dorsal striatum (caudate-putamen, CPU) and globus pallidus (GP). White matter tracts such as the corpus callosum (CC) are unlabeled. **c** Moderately dense staining is found in the olfactory bulb (OB). **d** Moderately dense staining is found in the prefrontal cortex (PFC). III indicates layer III. **e** Dense staining is found in the form of puncta in the neuropil, in the caudate-putamen (CPU). **f** Light labeling is observe in the hippocampus. CA1, field CA1; SP, stratum pyramidale (SP). **g** Light labeling is found in the thalamus (TH). **h** Dense labeling is found in the dorsal horn (DH) of the spinal cord. Scale: **a**, **c**–**h** = 200 μm. **b** = 2 mm

The cellular distribution of nSMase2 was next elucidated (Fig. 4). Control sections incubated with PBS instead of primary antibody showed absence of immunostaining (Fig. 4a), while parts of the brain were labeled in sections incubated with nSMase2 antibody (Fig. 4b). Dense labeling was observed in the striatum, including the caudate-putamen (Fig. 4b, e), while moderately dense staining was found in the olfactory bulb (Fig. 4c) and cerebral neocortex (Fig. 4d). The hippocampus (Fig. 4f) and thalamus (Fig. 4g) were lightly stained. Dense staining was found in the cochlear nuclei and dorsal horn of the spinal cord (Fig. 4h). The labeling pattern took the form of puncta in the neuropil, and cell outlines were indistinct. White matter tracts were very lightly labeled or unlabeled.

**Fig. 5:**
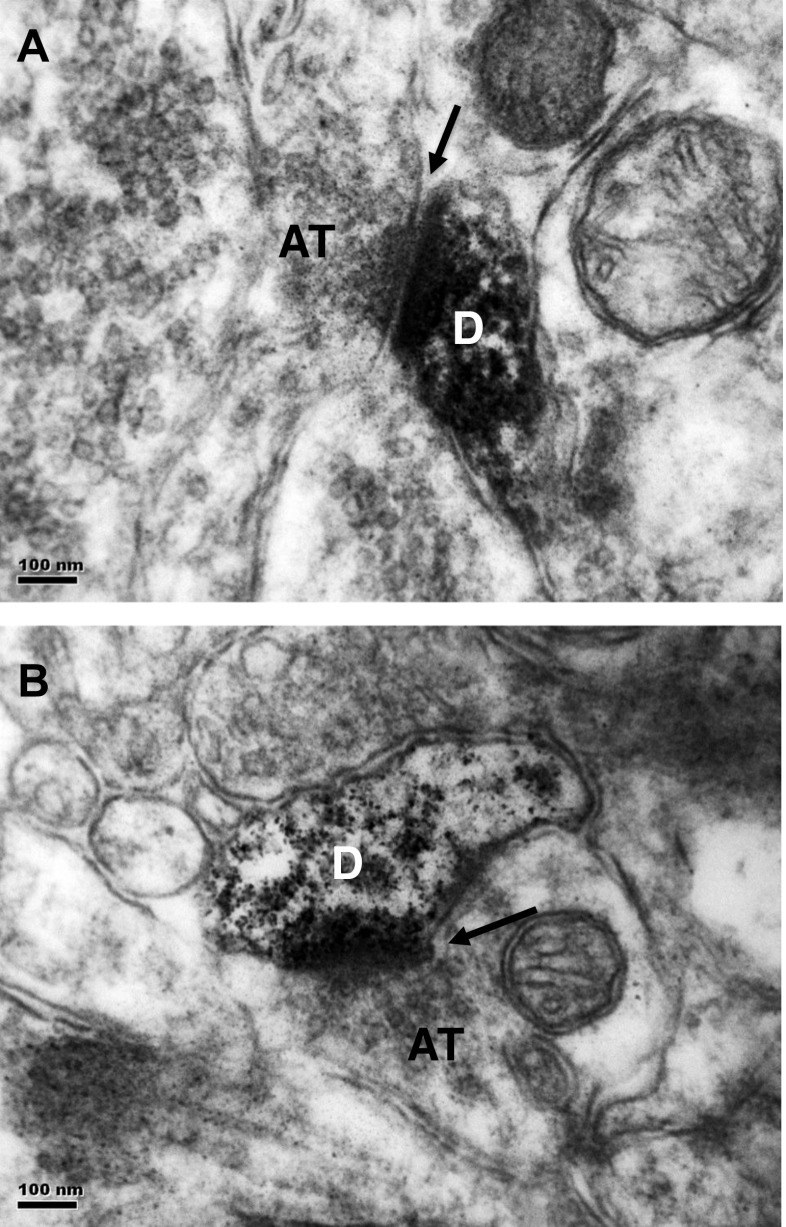
**a**, **b** Electron micrographs of nSMase2-immunolabeled sections of the striatum. Immunoreaction product is present in small diameter dendrites or dendritic spines (D) which forms asymmetrical synapses (arrows) with unlabeled axon terminals (AT). The dendrites are putatively identified as those of medium spiny neurons in the striatum, and axon terminals, as glutamatergic axons that project from the motor cortex. Scale = 100 nm

The subcellular localization of nSMase2 was further examined (Fig. 5). Electron microscopy of nSMase2-immunolabeled sections of the caudate-putamen showed that immunoreaction product was present in small diameter dendrites or dendritic spines, that formed asymmetrical synapses with unlabeled axon terminals containing small round vesicles, and characteristics of glutamatergic axons (Fig. 5a, b). The dendrites are putatively identified as those of medium spiny neurons in the caudate-putamen, and the axon terminals, as that of glutamatergic axons from the motor cortex.

### Alteration of Striatal Sphingolipids Due to Intrastriatal Inhibition of nSMase

**Fig. 6:**
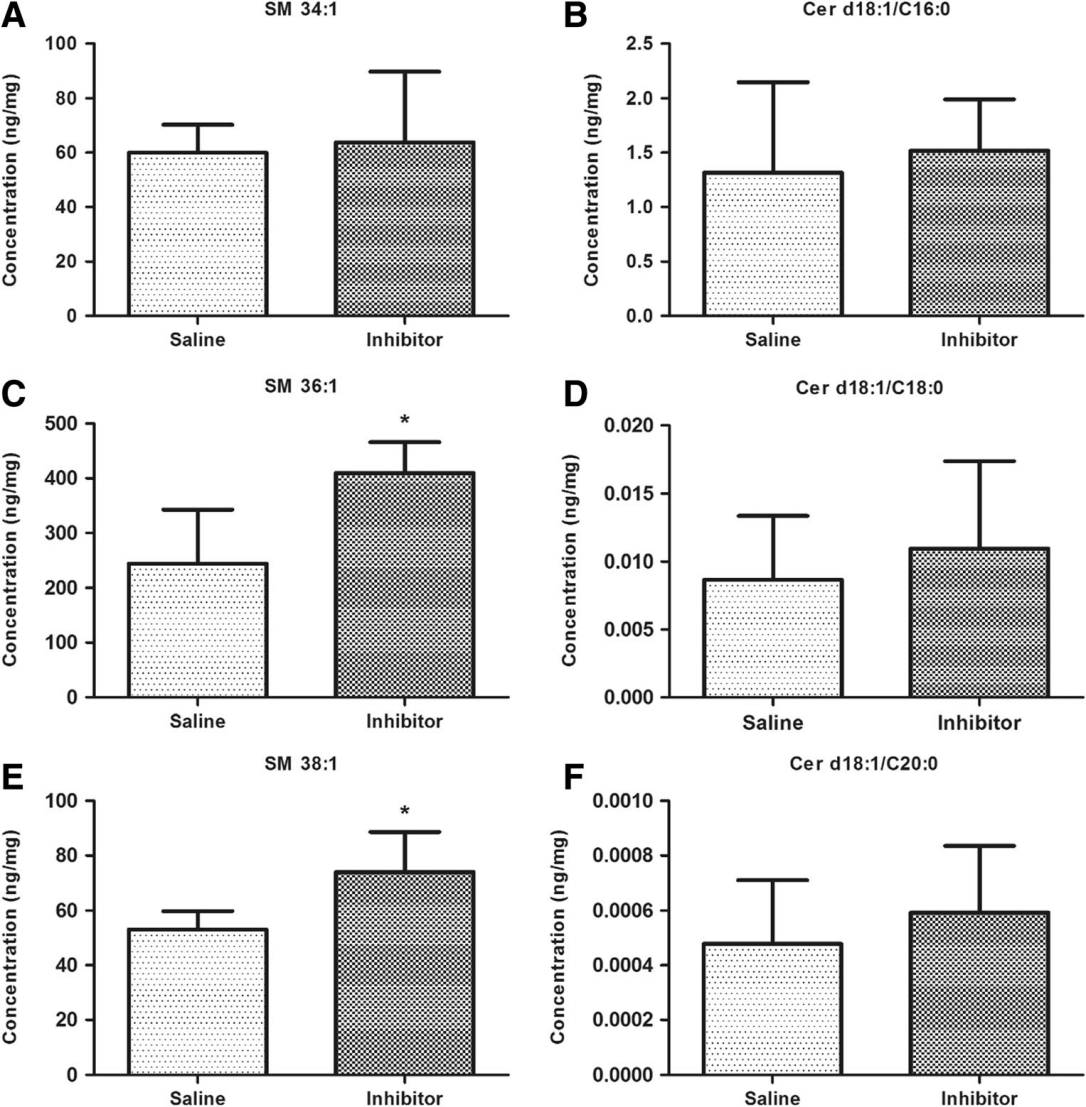
Lipidomic analysis of lipid raft fractions. **a**, **b** Quantitation of 34:1 sphingomyelin and its corresponding ceramide species. There is no significant difference between vehicle- and GW4869 nSMase inhibitor-treated groups. **c**–**f** Quantitation of longer chain 36:1 and 38:1 sphingomyelins and their corresponding ceramides. Sphingomyelins, but not ceramides, were significantly reduced by inhibitor treatment. Asterisks indicate significant differences compared to controls *p < 0.05

Lipidomic analysis showed significant increases in long chain sphingomyelin species 36:1 and 38:1 after intrastriatal injection of GW4869 indicating effectiveness of the inhibitor (Fig. 6). However, there was an absence of change in their corresponding ceramides. There was also an absence of change in another sphingomyelin species 34:1 (Fig. 6). Results showed that nSMase inhibition causes accumulation in certain long chain sphingomyelin species, but does not affect total ceramide levels.

### Alteration of Striatal Lipid Raft Proteome after Intrastriatal Inhibition or Knockdown of nSMase2

A total of 1423 high-confidence proteins were identified by proteomic profiling, with multiple peptides and < 1% FDR. We used a cutoff ratio value of 0.5 for downregulated and ratio value of 2 as upregulated proteins, i.e., 2-fold change. We identified a total of 269 downregulated proteins in lipid rafts of the striatum after nSMase2 antisense injection to the caudate-putamen, while a total of 383 proteins were downregulated after GW4869 injection. The common downregulated proteins in both treatment groups are listed in Table 3. Surprisingly, no common upregulated protein was identified. Results are consistent with the notion that nSMase2 activity is important for the aggregation or clustering of proteins in lipid rafts. A protein, annexin A6, was downregulated by 9-fold and 16-fold in the nSMase2 antisense and inhibitor injection group, respectively. Annexin 6 is present in lipid rafts of synaptic membranes [35]. It regulates plasma membrane remodeling of lipid rafts [36] and acts as a scaffold to link membrane microdomains with the cytoskeleton [37]. Other proteins that showed decreases after nSMase2 inhibition or antisense treatment include hippocalcin, which has been localized to Lewy bodies [38], N-myc downstream-regulated gene 1 (NDRG1), and carbonyl reductase 1.

**Table 3:** Proteins decreased in lipid raft fractions of rats injected with GW4869 nSMase inhibitor (Inb) or saline (Sal); or antisense (AS) vs. scrambled sense (SS)

Gene symbol	Name	Pro_Mass	AS/SS	Inb/Sal
Eef2	Elongation factor 2	96192	0.082391	0.356
Anxa6	Annexin	76108	0.105078	0.061913
Prdx5	Isoform cytoplasmic + peroxisomal of peroxiredoxin-5, mitochondrial	17195	0.115124	0.253253
Lonp1	Lon protease homolog, mitochondrial	106296	0.139431	0.267
Aars	Alanine-tRNA ligase, cytoplasmic	107522	0.139431	0.4005
Hpca	Neuron-specific calcium-binding protein hippocalcin	22527	0.174288	0.205385
LOC100361259	60S ribosomal protein L13	24168	0.179974	0.492118
Ctnna1	Catenin (cadherin-associated protein), alpha 1	100858	0.18126	0.4984
Spr	RCG56371	28510	0.185908	0.34176
Ssrp1	FACT complex subunit SSRP1	81206	0.185908	0.438154
Ndrg1	Protein NDRG	43383	0.193344	0.167529
Anxa7	Annexin	50272	0.2014	0.237333
Cbr1	Carbonyl reductase [NADPH] 1	30920	0.210555	0.248121
Coro1c	Coronin	53828	0.220765	0.328615
Dpp6	Dipeptidyl aminopeptidase-like protein 6	91842	0.2226	0.412211
Celf2	CUGBP Elav-like family member 2	54291	0.231018	0.397882
Oxr1	Oxidation resistance protein 1	97418	0.232385	0.323636
Gng3	Guanine nucleotide-binding protein subunit gamma	8527	0.250224	0.294869
H3f3b	Histone H3	15376	0.255078	0.441192
A0A0G2JTG1	Uncharacterized protein	9382	0.256959	0.286437
Park7	Park7 protein	23002	0.260142	0.44144
Tsg101	Tumor susceptibility gene 101	44221	0.264338	0.3115
Fkbp1a	Peptidyl-prolyl cis-trans isomerase FKBP1A	11972	0.270931	0.462391
Frrs1l	DOMON domain-containing protein FRRS1L	32892	0.271452	0.314986
Hist1h4b	Histone H4	11360	0.27173	0.416595
Pabpc1	Polyadenylate-binding protein 1	60149	0.27189	0.408525
Rpl24	60S ribosomal protein L24	17779	0.273329	0.196058
Chchd6	MICOS complex subunit Mic25	29592	0.2736	0.449684
A0A0G2KBA1	Uncharacterized protein	31964	0.274636	0.319673
Erp29	Endoplasmic reticulum resident protein 29	28614	0.274636	0.323636
Pitpna	Phosphatidylinositol transfer protein alpha isoform	32059	0.276925	0.209412
Actn3	Actin in alpha 3, isoform CRA_a	103575	0.278862	0.4272
Lancl2	LanC lantibiotic synthetase component C-like 2 (bacterial)	51677	0.278862	0.4628
Ube2n	Ubiquitin-conjugating enzyme E2 N	17170	0.281023	0.331163
Ndufs7	NADH dehydrogenase (ubiquinone) Fe-S protein 7	24215	0.28196	0.212085
Blvrb	Biliverdin reductase B (flavin reductase (NADPH))	22194	0.283219	0.33375
Dynll2	Dynein light chain 2, cytoplasmic	10457	0.283631	0.366812
Atp5j2	ATP synthase subunit f, mitochondrial	10503	0.283934	0.368729
Rab7a	Ras-related protein Rab-7a	21361	0.284329	0.440145
Sdhb	Succinate dehydrogenase [ubiquinone] iron-sulfur subunit, mitochondrial	32607	0.287714	0.090127
Canx	Calnexin	67612	0.287714	0.237333
Pdxp	Pyridoxal phosphate phosphatase	33380	0.287714	0.339048
Cdc42	Cell division control protein 42 homolog	21696	0.292945	0.146051
Cars	Cysteinyl-tRNA synthetase (predicted), isoform CRA_b	86110	0.3021	0.237333
Cacnb2	Calcium channel, voltage-dependent, beta 2 subunit, isoform CRA_d	68596	0.3021	0.237333
Ppp2r5b	Protein LOC100909468	57746	0.3021	0.34176
Lrrc7	Leucine-rich repeat-containing protein 7	168690	0.3021	0.356
Ddx5	DEAD (Asp-Glu-Ala-Asp) box polypeptide 5	69709	0.3021	0.356
Atad3	ATPase family AAA domain-containing protein 3	66889	0.3021	0.356
Gad2	Glutamate decarboxylase 2	66215	0.3021	0.356
Sacm1l	Phosphatidylinositide phosphatase SAC1	67509	0.3021	0.356
Clip2	CAP-Gly domain-containing linker protein 2	115975	0.3021	0.356
Aldh2	Aldehyde dehydrogenase, mitochondrial	56994	0.314688	0.303787
Uqcr10	Protein Uqcr10	7095	0.318671	0.150211
Pdia3	Protein disulfide-isomerase	57499	0.3192	0.322415
Suclg1	Succinate-CoA ligase, GDP-forming, alpha subunit, isoform CRA_b 1	37935	0.336958	0.4628
Pacsin1	Protein kinase C and casein kinase substrate in neurons protein 1	50760	0.342924	0.201217
Atp5o	ATP synthase subunit O, mitochondrial	23440	0.344484	0.277985
Btbd8	Protein Btbd8	149108	0.345257	0.4005
Rpl30	60S ribosomal protein L30	12947	0.355412	0.418824
Idh3B	Isocitrate dehydrogenase [NAD] subunit beta, mitochondrial	42612	0.358744	0.459717
Hnrnpab	CArG-binding factor A	30948	0.359643	0.32752
Ube2o	Protein Ube2o	126509	0.36252	0.4272
Pcdh1	Protein Pcdh1	129114	0.36252	0.4272
Fam49a	Family with sequence similarity 49, member A	37704	0.366182	0.2581
Cox6c2	Cytochrome c oxidase subunit 6C-2	8449	0.368122	0.279335
Cryab	Alpha-crystallin B chain	20076	0.368664	0.434441
Hspa5	78-kDa glucose-regulated protein	72473	0.369559	0.42417
Sfpq	Protein Sfpq	75210	0.372804	0.188075
Gsta1	Glutathione S-transferase	25360	0.375947	0.3115
Rac1	Ras-related C3 botulinum toxin substrate 1	24636	0.384326	0.229125
Pip4k2b	Phosphatidylinositol 5-phosphate 4-kinase type-2 beta	47633	0.384491	0.453091
Dnm1l	Dynamin-1-like protein	84369	0.387066	0.460283
Ccsap	Protein Ccsap	28584	0.387308	0.34176
Cct5	T-complex protein 1 subunit epsilon	59955	0.390953	0.326333
Ehd3	EH domain-containing protein 3	60810	0.390953	0.403467
Scrn1	Secernin-1	46994	0.394043	0.1246
Lynx1	Ly6/neurotoxin 1 (predicted), isoform CRA_a	13396	0.395996	0.466649
P11517	Hemoglobin subunit beta-2	16086	0.4028	0.165568
Map4	Microtubule-associated protein	235075	0.4028	0.2848
Hnrnpm	Isoform 2 of heterogeneous nuclear ribonucleoprotein M	56864	0.4028	0.474667
Wdr13	Protein Wdr13	43571	0.4028	0.258909
Epn2	Epsin 2	68809	0.4028	0.474667
Atp1a2	Sodium/potassium-transporting ATPase subunit alpha-2	113457	0.415242	0.495857
LOC684681	Protein LOC684681	21304	0.415388	0.109045
Pabpc4	Polyadenylate-binding protein	71188	0.42294	0.459355
Atp4a	Sodium/potassium-transporting ATPase subunit alpha	115674	0.435024	0.4272
Mog	Myelin-oligodendrocyte glycoprotein	28153	0.441366	0.363574
Nono	Non-POU domain-containing octamer-binding protein	55005	0.441531	0.449684
Phb2	Prohibitin-2	33148	0.444169	0.452486
Rhob	Rho-related GTP-binding protein RhoB	22565	0.447108	0.37024
Ldha	L-lactate dehydrogenase	36712	0.447414	0.415081
Syncrip	Heterogeneous nuclear ribonucleoprotein Q	62861	0.447941	0.41296
Rtn4rl2	Reticulon-4 receptor-like 2	46896	0.448277	0.260488
Gstm3	Glutathione S-transferase Yb-3	25835	0.45861	0.24935
Atp6v1c1	V-type proton ATPase subunit C 1	44044	0.459392	0.388364
Palm	Paralemmin-1	42072	0.459717	0.356
Idh3a	Isocitrate dehydrogenase [NAD] subunit, mitochondrial	41606	0.462791	0.33802
Acat1	Acetyl-CoA acetyltransferase, mitochondrial	45009	0.464422	0.458576
Rala	Ras-related protein Ral-A	23709	0.468255	0.4094
Rpl10a	60S ribosomal protein L10a	24987	0.469334	0.439319
Msn	Moesin	67899	0.469933	0.263704
Dlg2	Disks large homolog 2	95331	0.474729	0.3204
Fam49b	Fam49b protein	37038	0.477949	0.251655
Alb	Serum albumin	70682	0.48336	0.246206
Tppp	Protein Tppp	25738	0.48336	0.362473
Mapk1	Mitogen-activated protein kinase 1	41648	0.48336	0.4895

Proteins that were reduced by more than 50% by both genetic and pharmacological inhibition of nSMase2 are listed

### Rotarod Test

**Fig. 7:**
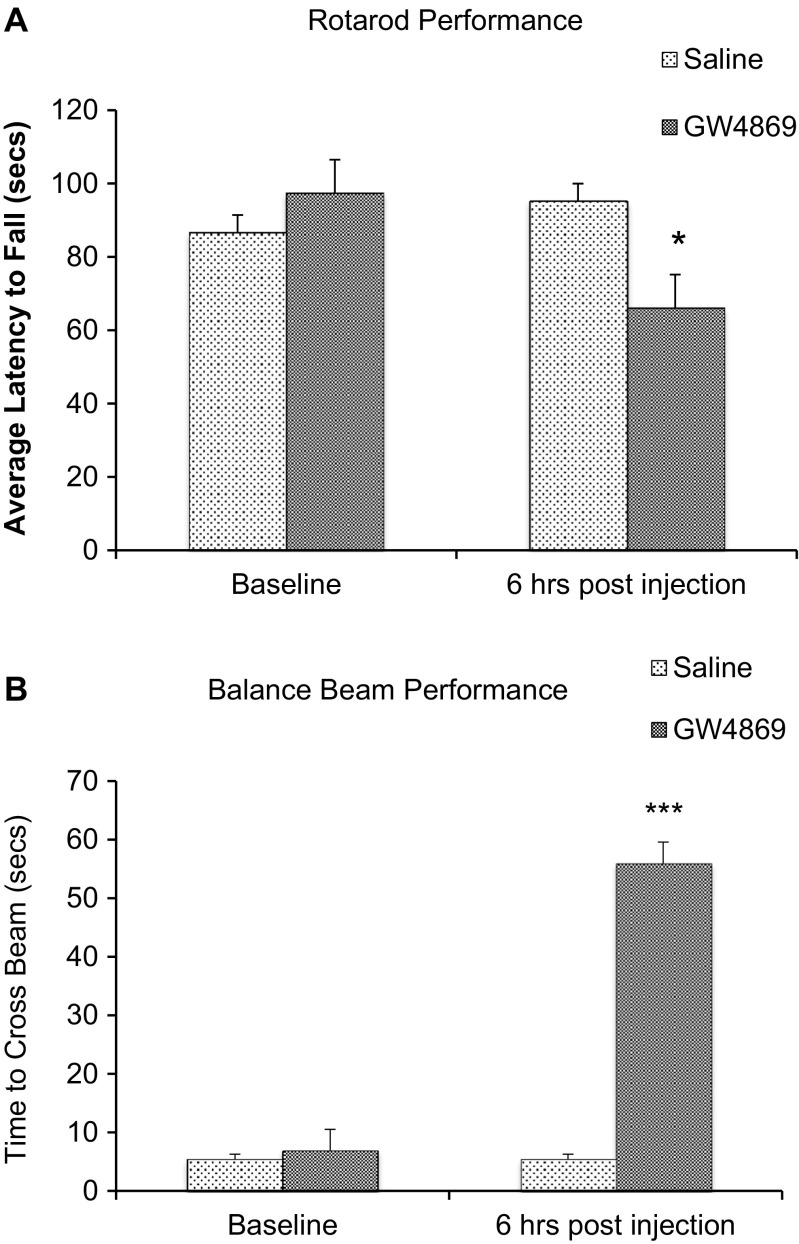
**a** Rotarod performance (saline vs GW4869 nSMase inhibitor). Histograms represent the time spent by rats injected with saline and rats injected with GW4869 walking on the accelerating rotating rod. Animals were tested 6-h post intrastriatal injection. Data represents the mean and standard error from *n* = 6 Wistar rats in each group. Each bar in the diagram indicates mean + SEM. **b** Beam crossing test (saline vs inhibitor). Histograms represent the time spent by rats injected with saline and rats injected with GW4860 to cross the beam. Rats that fell off the beam or were unable to cross the beam successfully after 60 s were given a maximum score of 60 s. Animals were tested 6-h post intrastriatal injection. Data represents the mean and standard error from *n* = 6 Wistar rats in each group. Each bar in the diagram indicates mean + SEM. Asterisks indicate significant differences compared to controls: *p < 0.05, **p < 0.01, ***p < 0.001

**Fig. 8:**
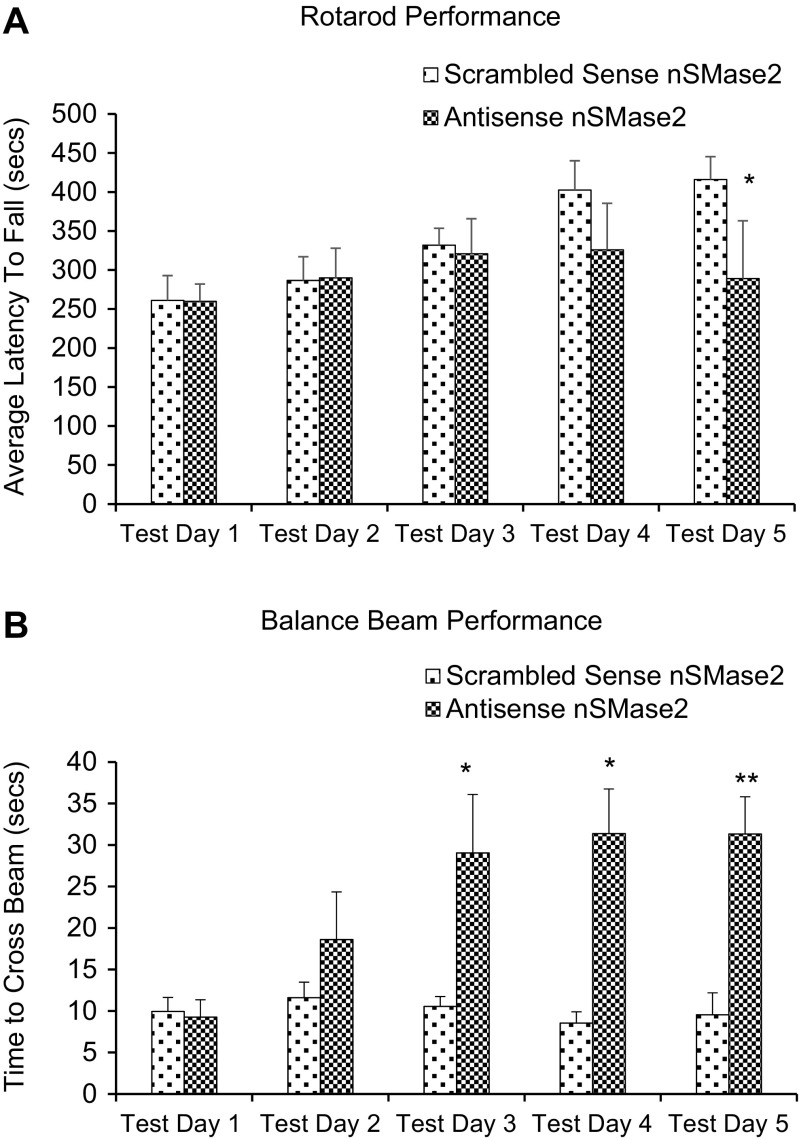
**a** Rotarod performance (scrambled sense vs antisense). The histograms represent the time spent by rats injected with scrambled sense nSMase2 and rats injected with antisense nSMase2 walking on the accelerating rotating rod. Animals were tested for 5 days post intrastriatal injection. Data represents the mean and standard error from *n* = 6 Wistar rats in each group. Each bar in the diagram indicates mean + SEM. **b** Beam crossing test (scrambled sense vs antisense). The histograms represent the time spent by rats injected with saline and rats injected with GW4860 to cross the beam. Rats that fell off the beam or were unable to cross the beam successfully after 60 s were given a maximum score of 60 s. Animals were tested for 5 days post intrastriatal injection. Data represents the mean and standard error from *n* = 6 Wistar rats in each group. Each bar in the diagram indicates mean + SEM. Asterisks indicate significant differences compared to controls: *p < 0.05, **p < 0.01, ***p < 0.001

Rats injected with GW4869 in the striatum remained on the rotarod for a significantly shorter period of time, as compared to saline-injected controls (*p <* 0.05) (Fig. 7a). Likewise, rats injected with antisense nSMase2 oligonucleotide remained on the rotarod for a significantly shorter period of time, as compared to scrambled sense-injected controls (*p <* 0.05) (Fig. 8a).

### Beam Crossing Test

Rats exhibited poorer performance on the beam crossing test after intrastriatal injection of GW4869. These were unable to place both hind paws on the horizontal surface of the beam but attempted to balance and traverse the beam by placing their forepaw on the horizontal surface and dragging themselves across the beam, before losing balance and falling off the beam. They were unable to successfully cross the beam in all except one trial (Fig. 7b). Rats injected with vehicle control showed no significant difference in performance before and after injection. There was no significant difference in time taken to cross the beam, between the two groups, before injection. Rats injected with antisense oligonucleotides to nSMase2 showed significantly poorer performance in the balance beam test, compared to scrambled sense-injected controls (*p <* 0.05) (Fig. 8b).

### Acoustic Startle Reflex Test and Prepulse Inhibition of Acoustic Startle

**Fig. 9:**
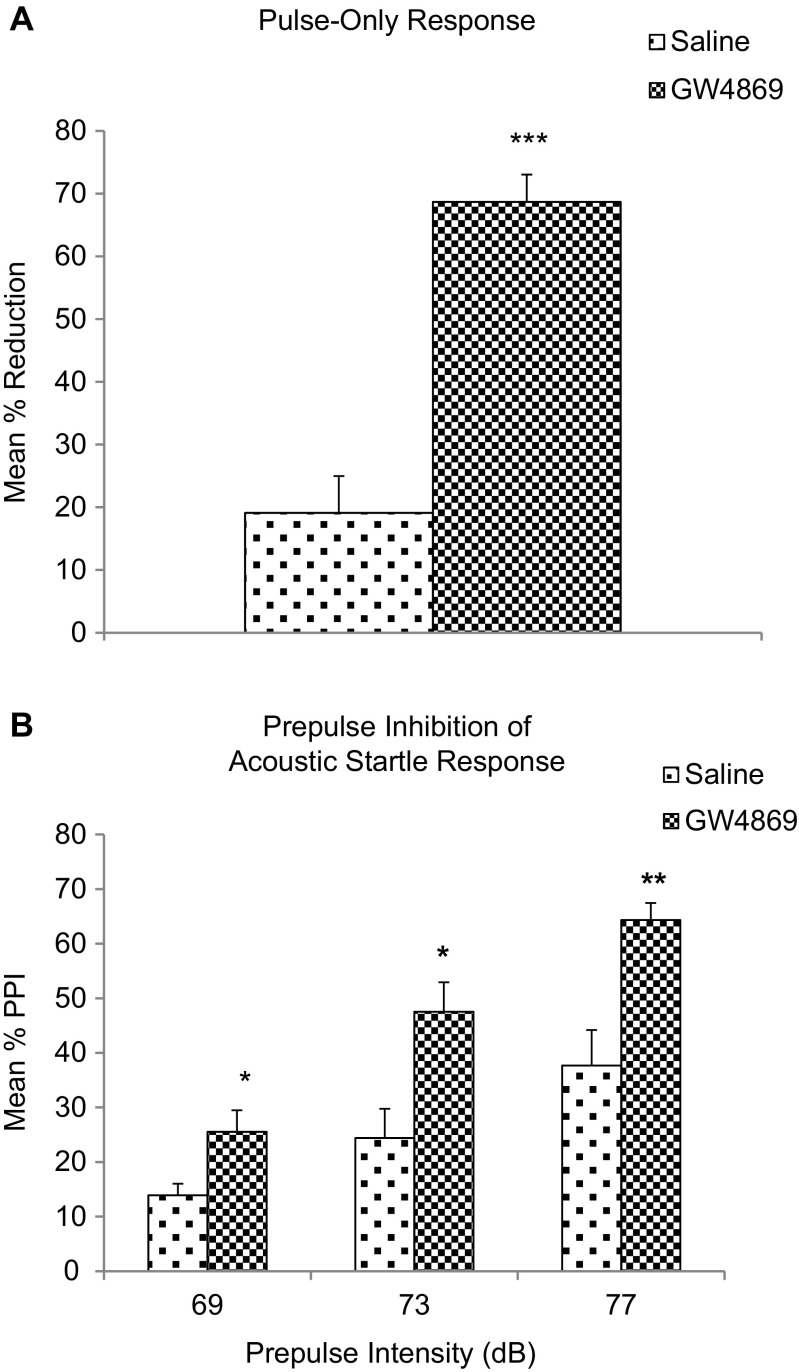
**a** Acoustic startle reflex. Mean percentage differences in auditory startle response before treatment and after intrastriatal saline or GW4869 injection. The acoustic startle response test was conducted 6 h after saline or GW4869 intrastriatal injection. Data represents the mean and standard error from *n* = 6 Wistar rats in each group. Each bar in the diagram indicates mean + SEM. **b** Prepulse inhibition. Prepulse inhibitions of the auditory startle response at three prepulse intensities after saline or GW4869 administration. The acoustic startle response test was conducted 6 h after saline or GW4869 intrastriatal injection. Data represents the mean and standard error from *n* = 6 Wistar rats in each group. Each bar in the diagram indicates mean + SEM. Asterisks indicate significant differences compared to controls: *p < 0.05, **p < 0.01, ***p < 0.001

The amplitude of the acoustic startle response was first compared using pulse-alone trials. Rats that received GW4869 injection showed significantly decreased acoustic startle response compared to saline-injected controls (Fig. 9a). Prepulse inhibition (PPI) of the auditory startle response was also determined after GW4869 or saline injection. Rats that received GW4869 injection showed significantly increased PPI as compared to saline-injected controls (Fig. 9b).

### TUNEL and DAPI Histochemistry

GW4869 treatment did not cause an increase in nuclear fragmentation in the TUNEL assay, while positive control sections displayed TUNEL-positive staining of the nuclei. Results imply that injected treatments did not cause the induction of cell death in the striatum (Supplementary Figure).

## Discussion

nSMase2 is the most well-studied mammalian nSMase of the sphingomyelinase family and has been shown to be mainly found in the brain [7, 39, 40]. Our real-time RT-PCR results showed that nSMase2 mRNA is present in different brain regions, with the highest expression in the striatum, followed by the cerebral cortex and low expression in the brainstem and cerebellum. Western blots showed high levels of nSMase2 protein expression in the striatum and cortex and low expression in the brainstem and cerebellum, which is consistent with the RT-PCR results. The 71-kDa band detected is in accordance with the molecular weight of full length nSMase2 [4] and was significantly reduced by antisense oligonucleotide knockdown in the striatum, indicating specificity of the antibody. Immunohistochemical analyses revealed dense staining in the striatum, cochlear nuclei, and dorsal horn of the spinal cord, and moderate staining in cerebral neocortex. White matter tracts such as the corpus callosum were very lightly labeled or unlabeled. The above results are in agreement with previous findings that nSMase activity is highest in the striatum among different regions in the human and rat brain—with activity in the stratum being 2.7 times greater than that of the parietal cortex, and 10 times that of the cerebellum, brainstem, and corpus callosum [28, 39]. Although our real-time RT-PCR results reveal high nSMase3 expression across all brain regions including the brainstem and cerebellum, previous studies that measured nSMase enzymatic activity have failed to detect this trend [28]. nSMase3 does not share any sequence homology or catalytic core residues [8] and its activity may differ from other nSMases [34]. It is possible that nSMase3 may have a different function from other isoforms in the brain.

Lipidomic analysis showed that SM36:1 and SM38:1 were increased after nSMase was blocked in the striatum, indicating effectiveness of the GW4869 nSMase inhibitor. However, there was an absence of change in another sphingomyelin species 34:1, consistent with previous reports that nSMase2 exhibits substrate preference for longer chain sphingomyelins [41]. Interestingly, an expected decrease in corresponding 36:1 and 38:1 ceramides was not observed. Results indicate that nSMase inhibition causes accumulation in certain sphingomyelin species by preventing breakdown into their corresponding ceramides. The unchanged ceramide levels after nSMase inhibition may be due to compensation from de novo sphingolipid synthesis, which is a major source of ceramide generation and the only source of non-dietary sphingolipids [42]. These findings suggest that sphingomyelin accumulation is the primary cause of the observed phenotype, and not the depletion of ceramides per se. Proteomic analyses showed significant decreases in many proteins due to inhibition or antisense knockdown of nSMase2 in the striatum. This could have functional consequences due to changes in aggregation or clustering of these proteins. One of the proteins that showed decreases after antisense knockdown or inhibition of nSMase2 is annexin A6. This is a member of the annexin family of calcium-dependent membrane and phospholipid-binding proteins, which creates a scaffold for the formation of multifactorial signaling complexes and functions as an organizer of membrane domains to modulate intracellular cholesterol homeostasis, and regulates transient membrane-actin interactions during endocytic and exocytic transport [37, 43].

The high level of expression of nSMase2 in the striatum suggests that the enzyme may be important in the function of this brain region. We investigated the involvement of nSMase2 in motor function and coordination via intrastriatal injection of GW4869 a well-established specific inhibitor of nSMase2 that does not affect A-SMase activity [12, 23, 44–49], or antisense oligonucleotide to nSMase2. Rats that received intrastriatal injection of GW4869 or antisense oligonucleotide to nSMase2 showed impaired motor function in the rotarod test, and remained on the rotarod for a shorter duration compared to controls. Similarly, rats injected with GW4869 or antisense oligonucleotide displayed poorer motor coordination in the beam crossing test, with all rats falling off the beam and unable to successfully cross the beam. Results indicate an important role of nSMase2 in regulation of motor activity and coordination [50]. A role of nSMase2 in the startle reflex and prepulse inhibition of startle was also investigated. Inhibition of nSMase2 in the striatum resulted in decreased acoustic startle response and increased prepulse inhibition (%PPI) compared to controls, indicating an improvement in sensorimotor gating. Besides the caudate-putamen or dorsal striatum, it is possible that nSMase2 could have a role in the ventral striatum and the reward pathway, which extends from the ventral tegmental area to the nucleus accumbens shell [51]. This possibility needs to be investigated in future studies.

The exact molecular mechanisms of how nSMase2 plays a role in the regulation of striatal activity are not fully understood. Nevertheless, our EM finding that nSMase2 was present in small diameter dendrites or dendritic spines of asymmetrical synapses with unlabeled axon terminals that contained small round vesicles, and features of glutamatergic axons [52, 53], suggests that it may affect excitatory neurotransmission. Lipid rafts are docking sites for glutamate receptors [54–56], and nSMase2 has been shown to regulate AMPA receptor numbers and NMDA glutamate receptor subunit composition and clustering [22, 23]. Long-term inhibition of nSMase2 increases PSD-95, as well as the amount of NMDA receptor NR2A subunits and AMPA receptor GluR1 subunits [23]. Knockout of the AMPA receptor subunit GluR1 results in increased level of dopamine in the striatum [57] and increased locomotion [58]. We postulate that nSMase2 could have a role in modulating glutamatergic transmission, and that enzyme inhibition could result in alterations in excitatory transmission at the corticostriatal synapse and decreased motor function. A change in excitatory transmission could also be consistent with the observed improvement in prepulse inhibition of the auditory startle response. Further work is necessary to determine the molecular mechanisms of nSMase2 action in the brain, and possible changes in enzyme activity in neurological and psychiatric disorders.

## Electronic supplementary material

Supplementary Figure Detection of apoptosis after intrastriatal injection of nSMase2 inhibitor GW4869 and saline. A-C Positive control. (A DAPI staining (blue) B TUNEL assay (green) C merged image of TUNEL and DAPI counterstaining (green-blue)). D-F Negative Control. G-I Saline injected sections. J-L GW4869 injected sections. No apparent DNA fragmentation (column 2, TUNEL staining) was detected except in positive controls. Scale bar: 200 μm. (PPTX 1267 kb)

## Abbreviations

Aβ: amyloid beta
AD: Alzheimer’s disease
AMPA: α-amino-3-hydroxy-5-methyl-4-isoxazolepropionic acid
DAPI: 4′,6-diamidino-2-phenylindole
LC-MS/MS: liquid chromatography-mass spectrometry/mass spectrometry
NMDA: *N*-methyl-d-aspartate
nSMase2: neutral sphingomyelinase 2
PD: Parkinson’s disease
PMA: phorbol 12-myristate 13-acetate
PVDF: polyvinylidene difluoride
SDS-PAGE: sodium dodecyl sulfate polyacrylamide gel
siRNA: small interfering RNA
TBS-Tween: Tris-buffered saline—Tween
TNF-α: tumor necrosis factor-alpha
TUNEL: terminal deoxynucleotidyl transferase dUTP nick end labeling
