# Supplementary material for: Enriched Expression of Neutral Sphingomyelinase 2 in the Striatum is Essential for Regulation of Lipid Raft Content and Motor Coordination
Source: Mol Neurobiol. 2017 Oct 17;55(7):5741–56. doi: 10.1007/s12035-017-0784-z (PMC5994222; doi:10.1007/s12035-017-0784-z)
Supplement: Supplementary file 1 — Detection of apoptosis after intrastriatal injection of nSMase2 inhibitor GW4869 and saline. A-C Positive control. (A DAPI staining (blue) B TUNEL assay (green) C merged image of TUNEL and DAPI counterstaining (green-blue)). D-F Negative Control. G-I Saline injected sections. J-L GW4869 injected sections. No apparent DNA fragmentation (column 2, TUNEL staining) was detected except in positive controls. Scale bar: 200 μm. (PPTX 1267 kb) [file 12035_2017_784_MOESM1_ESM.pptx]

## Slide 1
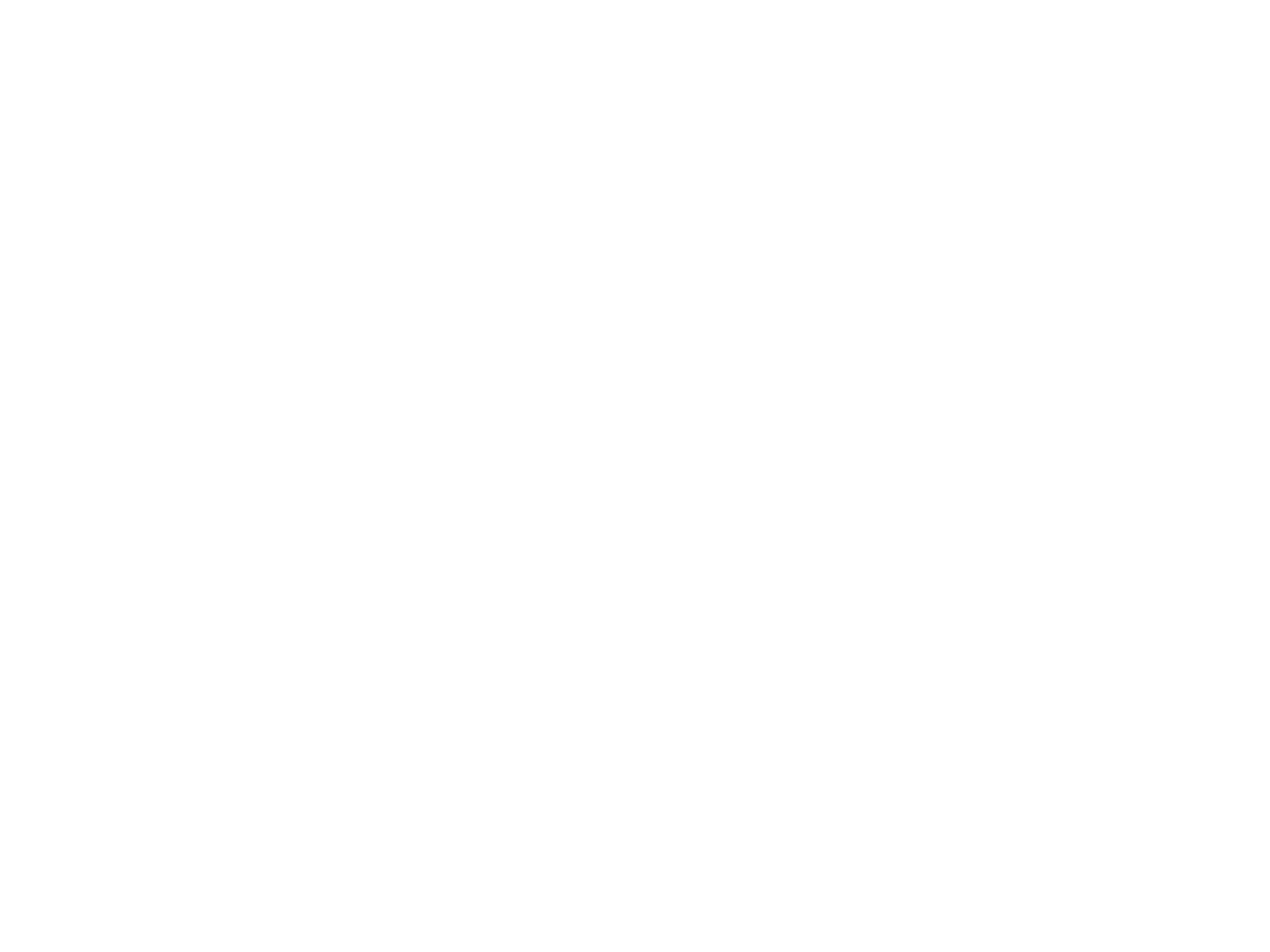

## Slide 2
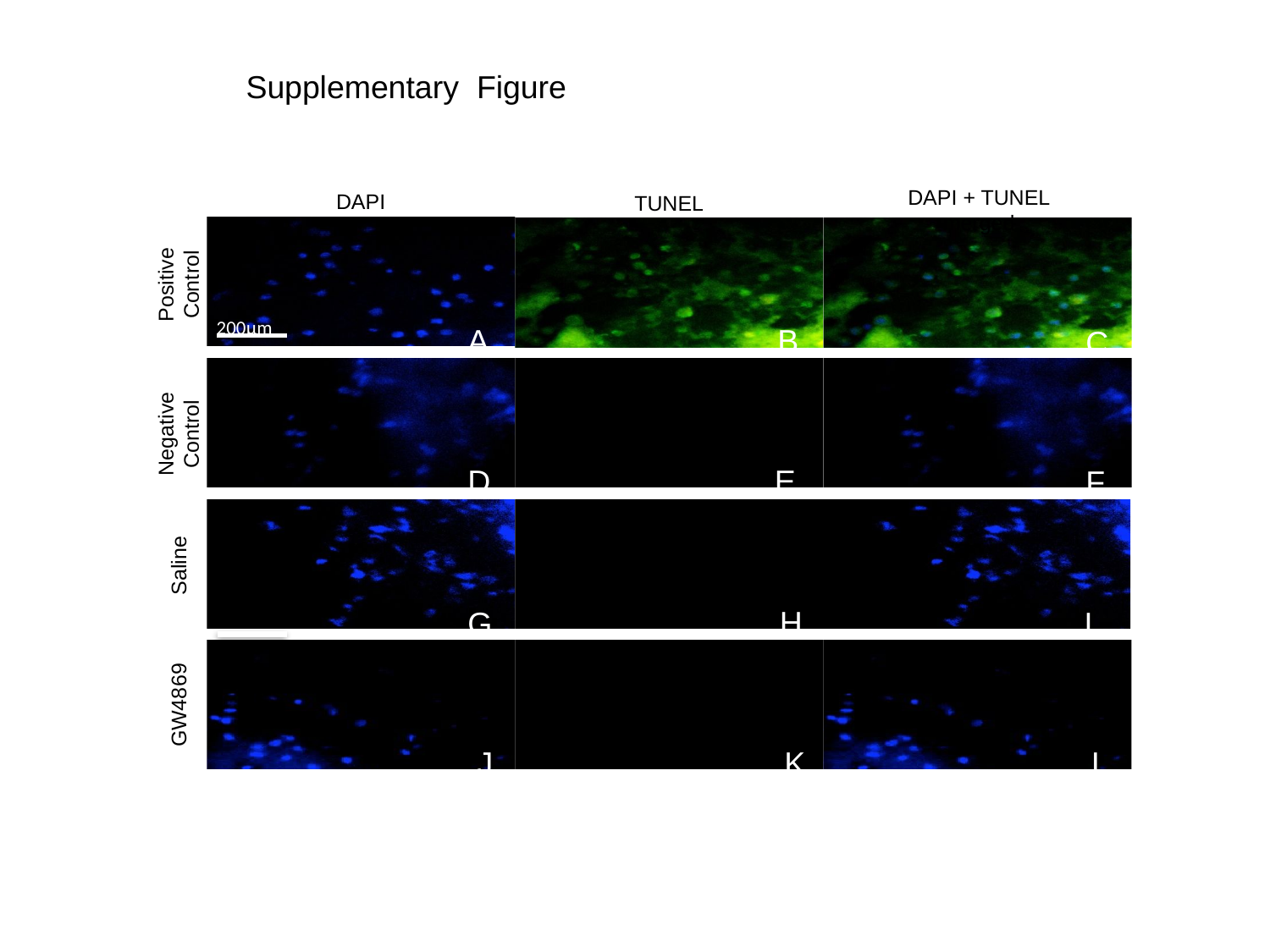

# Supplementary Figure
DAPI + TUNEL
merged
DAPI
TUNEL
Positive Control
A
B
C
Negative Control
D
E
F
Saline
H
G
I
GW4869
J
K
L
200μm
